# Prediction of organophosphorus insecticide-induced intermediate syndrome with stimulated concentric needle single fibre electromyography

**DOI:** 10.1371/journal.pone.0203596

**Published:** 2018-09-27

**Authors:** Chanika Alahakoon, Tharaka L. Dassanayake, Indika B. Gawarammana, E. Michael Sedgwick, Vajira S. Weerasinghe, Ahmed Abdalla, Michael S. Roberts, Nicholas A. Buckley

**Affiliations:** 1 Department of Physiology, University of Peradeniya, Peradeniya, Sri Lanka; 2 South Asian Clinical Toxicology Research Collaboration, University of Peradeniya, Peradeniya, Sri Lanka; 3 School of Psychology, The University of Newcastle, Newcastle, New South Wales, Australia; 4 Department of Medicine, University of Peradeniya, Peradeniya, Sri Lanka; 5 Basil Hetzel Institute for Translational Health Research, School of Pharmacy & Medical Sciences, University of South Australia, Adelaide, Australia; 6 Pharmaceutical Chemistry Department, Helwan University, Cairo, Egypt; 7 Therapeutics Research Centre, Diamantina Institute, University of Queensland, Queensland, Australia; 8 Translation Research Institute, Brisbane, Queensland, Australia; 9 The Discipline of Pharmacology, School of Medical Sciences, University of Sydney, Sydney, Australia; Weizmann Institute of Science, ISRAEL

## Abstract

**Background:**

Deliberate self-poisoning (DSP) using organophosphorus (OP) insecticides are a common clinical problem in Asia. OPs inhibit acetylcholine esterase (AChE), leading to over-activity of muscarinic and nicotinic cholinergic circuits. Intermediate syndrome (IMS) is mediated via prolonged nicotinic receptor stimulation at the neuromuscular junction and its onset is between 24–96 hours post ingestion. The aims of the present study were 1) to investigate whether neuromuscular junction dysfunction within the first 24 hours following exposure, quantified by jitter in single fibre electromyography (SfEMG), can predict IMS, and 2) to compare the changes in SfEMG jitter over the course of the illness among patients who developed IMS (IMS+) and those who did not (IMS-).

**Methods and findings:**

We conducted a prospective cohort study in a tertiary care hospital in Sri Lanka on 120 patients admitted between September 2014 and August 2016 following DSP by OP insecticides viz., profenofos 53, phenthoate 17, diazinon 13, chlorpyrifos 5, others 12, unknown 20. SfEMG was performed every second day during hospitalization. Exposure was confirmed based on the history and red blood cell AChE assays. IMS was diagnosed in patients who demonstrated at least three out of four of the standard IMS criteria: proximal muscle weakness, bulbar muscle weakness, neck muscle weakness, respiratory paralysis between 24–96 hours post ingestion. Respiratory failure requiring intubation occurred in 73 out of 120 patients; 64 of these were clinically diagnosed with IMS. Of the 120 patients, 96 had repeated SfEMG testing, 67 of them being tested within the first 24 hours. Prolonged jitter (>33.4μs) within the first 24 hours was associated with greatly increased risk of IMS (odds ratio = 8.9, 95% confidence intervals = 2.4–29.6, p = 0.0003; sensitivity 86%, specificity 58%). The differences in jitter between IMS+ and IMS- patients remained significant for 72 hours and increased jitter was observed in some patients for up to 216 hours. For intubated patients, the median time for jitter to normalize and median time to extubate were similar, and the two variables had a moderate positive correlation (r = 0.49, P = 0.001).

**Conclusions:**

Prolonged jitter recorded with SfEMG <24 hours of ingestion of an OP strongly correlates with subsequent occurrence of IMS. The time course of electrophysiological recovery of the NMJ was similar to the time course of respiratory recovery in IMS patients.

## Introduction

Self-poisoning carries a heavy disease burden in developing countries [1–3]. Ingestion of organophosphorus (OP) insecticides is a common method of deliberate self-harm in these countries [4, 5]. Despite recent regulations in Sri Lanka (and elsewhere) to reduce the availability of the most toxic pesticides, there are still very many hospital admissions, and the morbidity and case fatality remain high [6, 7].

OPs irreversibly inhibit acetylcholine esterase (AChE) enzyme in the synaptic cleft resulting in accumulation of acetylcholine. Excess acetylcholine initially stimulates the cholinergic receptors and subsequently leads to depolarizing block. Many of the features of cholinergic crisis in the initial period following ingestion are due to overstimulation of muscarinic receptors, and these effects can be overcome by atropine [8–11]. In 1987, Senanayake and Karalliedde reported an intermediate syndrome (IMS) of muscular paralysis observed in OP-poisoned patients with onset between 24–96 hours after the cholinergic features have subsided [12]. This weakness was seen first in neck muscles, followed by weakness of the muscles innervated by cranial nerves, proximal limb girdle muscles and the respiratory muscles. Many patients with IMS develop respiratory failure [13, 14].

The pathophysiology of IMS is yet to be fully understood. Sedgwick and Senanayake (1997) postulated that neuromuscular junctional blockage occurred due to the presence of persistently high levels of acetylcholine in the synaptic cleft, and this manifested as the IMS [15]. Subsequently, several case series and reports have described quantitative assessments of neuromuscular junctional transmission using electrophysiological methods, including repetitive nerve stimulation (RNS) [9, 16–20], and single fiber electromyography (SfEMG) [15, 21].

Assessment of the security of transmission at the neuromuscular junction using SfEMG was developed by Stålberg [22] and is used routinely for the diagnosis of neuromuscular disorders. The SfEMG focuses on measuring time for the junction to excite a muscle fibre between one excitation and the next. This normally varies slightly and is referred as ‘jitter’. Jitter is quantified as the mean consecutive difference (MCD) between one discharge and the next over a large number of repetitions. A stressed neuromuscular junction will show increased jitter. Further, SfEMG will measure when some impulses fail to cross the junction, a neuromuscular block. Clinical weakness is noted when around 20% of impulses are blocked [23].

In the present study, we aimed 1) to investigate whether neuromuscular junction dysfunction within the first 24 hours following exposure, quantified by jitter in single fibre electromyography (SfEMG), can predict IMS, and 2) to compare the changes in SfEMG jitter over the course of the illness among patients who developed IMS and those who did not.

## Methods

### Design and setting

A prospective cohort study was conducted over a 24-month period from September 2014 to August 2016 on all OP poisoned patients who were admitted to the Teaching Hospital Peradeniya (THP). The hospital is a tertiary care centre that receives both direct admissions and transfers from other hospitals of the Central Province of Sri Lanka. Ethical clearance for this study was granted by the Human Research Ethics Committee of the University of Peradeniya, Sri Lanka. Informed written consent was obtained prior to recruitment of all the participants. For those who were intubated or unconscious, consent was obtained from the next of kin, and informed written consent from the patients was taken later once they regained consciousness. For those patients who were under the age of 18, the written informed consent was obtained from parents or guardians.

### Participants

Admitted patients who had two or more cholinergic features (i.e. miosis, excessive sweating, impaired consciousness, fasciculations of limbs or tongue, bradycardia, lung crepitations, or respiratory rate < 12/minute) and reliable circumstantial evidence of ingesting an OP were included in the study. OP ingestion was based on the history obtained from the patient or a relative, the pesticide container, and/or documentation in the transferred medical records. The type of OP ingested by the patients were identified and was confirmed by analyzing the blood samples that were collected from the patients on admission. Red blood cell acetylcholinesterase (AChE) inhibition was estimated using Testmate ChE (EQM Research, Inc., Cincinnati, OH). This has been previously validated in Sri Lanka against a reference laboratory in Munich, Germany [24, 25]. No other biomarkers (e.g. neuropathy target esterase or paraoxonase polymorphism) were measured.

We excluded children less than 16 years of age, pregnant women and anyone with a history of previous neurological disease.

### Clinical assessment

All patients had a comprehensive neurological examination on admission to the hospital. Serial neurological assessments were done every 12 hours until the patient was discharged. Consistency of the clinical parameters that were observed was maintained by objective assessment, and recorded with the aid of a structured datasheet which included grading of muscle power. All clinical assessments were done by medically qualified clinical research assistants. All these assessments were then reviewed by the first author (CA). Patient management was conducted by a specialist physician (IBG).

A prospective definition for IMS was adhered to, based on the original description namely; weakness of bulbar muscles, proximal limb girdle and neck muscles usually observed between 24–96 hours and subsequent respiratory failure [12]. The weakness of proximal muscles and neck flexion was considered significant when the muscle power was grade 3 or less according to the Medical Research Council grading [14]. Respiratory failure was indicated by the need to ventilate the patient. While cholinergic features had usually subsided 24 hours after OP ingestion, short relapses of muscarinic signs or symptoms did not exclude the diagnosis. All IMS cases were confirmed by IBG who assessed the patients on daily basis as the consultant physician of the ward. He was blind to the SfEMG results of the patients.

### Routine management

Institutional practice is based on national guidelines of management of OP poisoning. Guidelines recommend an initial bolus of 0.6 (mild poisoning) to 3mg (severe poisoning). Boluses are repeated every 5-10minutes, each time doubling the previous dose. Once, patients are atropinized, a continuous infusion adjusted according to clinical features [10]. If signs of atropine overdose were observed (e.g., non-reactive dilated pupils, tachycardia etc.), the infusion rate was reduced or discontinued until they subsided. If patients developed further cholinergic features further boluses of atropine and readjustment of infusion was made. National guidelines do not recommend the use of oxime as clinical studies to date do not indicate any benefits [10, 26].

In patients who were given atracurium for intubation and ventilation, SfEMG testing was done at least 2 hours (i.e. after four half-lives) following the previous dose of atracurium, when the drug was assumed to be no longer effective. In patients who were sedated and ventilated with midazolam, which has no effect on the neuromuscular junction, SfEMG testing was not dependent on the dosing schedule.

### Stimulated concentric needle single fiber electromyography

Stimulated SfEMG recording were performed on the orbicularis oculi, which is among the first muscles to become weak after OP poisoning [27], with a fine concentric needle electrode using a portable Medelec Synergy EMG system (Medelec Synergy, Surrey, UK). Testing was done within 24 hours of ingestion, or for patients who were transferred to the study centre after 24 hours, at the earliest possible time point after admission, and thereafter every other day until the patient was discharged. All tests were performed by the first author (CA) at the bedside.

We followed as closely as possible the techniques previously described [23, 28] but with some variations. Stimulation was by a unifocal (monopolar) needle electrode inserted close to the suprazygomatic branch of the facial nerve. A disposable monopolar EMG needle electrode, 37mm long and 0.46mm diameter (TECA Natus neurology^®^ type 902-DMG37-TP) was the cathode, whereas a 10mm diameter EEG scalp electrode placed on the cheek was the anode. Another EEG electrode placed at the midline on the forehead (Fp position) served as the ground electrode. With the stimulating electrodes in place, stimulation at 2/s was used to find a location in the orbicularis oculi muscle where a twitch was evoked by a low stimulus current, less than 3 mA. The stimuli were delivered as rectangular pulses with widths in the range of 0.05–0.2 ms.

A concentric needle electrode (TECA Natus neurology^®^ S53153, 25mm x 0.30mm) with an oval recording surface of approximately 300 x 80 μm was inserted into the area of the muscle twitch in the orbicularis oculi muscle. Care was taken to always insert the needle in a direction away from the globe of the eye. Amplifier filter settings were 1000Hz for high pass and 10,000Hz for low pass. When a suitable signal was obtained we checked the stimulus threshold to be sure that stimulation was supra-threshold for the fibres seen. The stimulus rate was increased to 10/s and 100 traces were recorded. We aimed to record 20 fibres in each subject.

### SfEMG signal analysis

SfEMG recordings were subsequently analysed offline. The stimulated SfEMG software measured the jitter of the peak of the selected potential. Potentials are often referred to as ‘apparent single fibre potentials’ because, although they look like unitary single fibre events, there is always the possibility that they represent two or more overlapping potentials [29]. Records were scrutinized off-line to exclude this possibility before mean consecutive difference (MCD) measurements were taken [23]. Traces were examined at the fastest practical sweep speed and potentials meeting negative going potentials with a rise time of 0.5 milliseconds or less, potentials with a unitary appearance, that is without shoulders or notches and separated from any other potentials in the sweeps or potentials of amplitude greater than 100μV were included for analysis.

Excluded were positive going potentials which indicated damaged fibres. Potentials with notches or shoulders on the rising phase or which had an inconsistent shape were deemed to be composites of more than one fibre, and were rejected. Sometimes a potential appears single and unitary but, when all sweeps are examined, it is revealed that they are composites. Sweep superimposition and raster displays are useful for detecting composite potentials.

We looked for blocking of fibres with normal jitter; this signifies stimulation strength hovering around the threshold for that fibre. Such fibres were re-recorded after adjusting the stimulus strength or rejected if that was not possible. Sometimes a small increase in stimulus strength recruited additional fibres which could not be separated from one another.

Sudden jumps in latency indicate movement of the electrode or recruitment of another fibre. Sometimes two or more fibres were so close together in latency that they overlapped in the superimposed sweeps. Fibres showing these phenomena were excluded as their jitter could not be reliably measured. All other fibres whose jitter could be measured were included to minimize observer bias.

Stimulus was continuous at 10/sec so there were no effects of the velocity recovery function of the muscle fibre [30]. We feel confident that with long latency fibres we were not recording blink reflexes or F responses [31].

**Fig 1:**
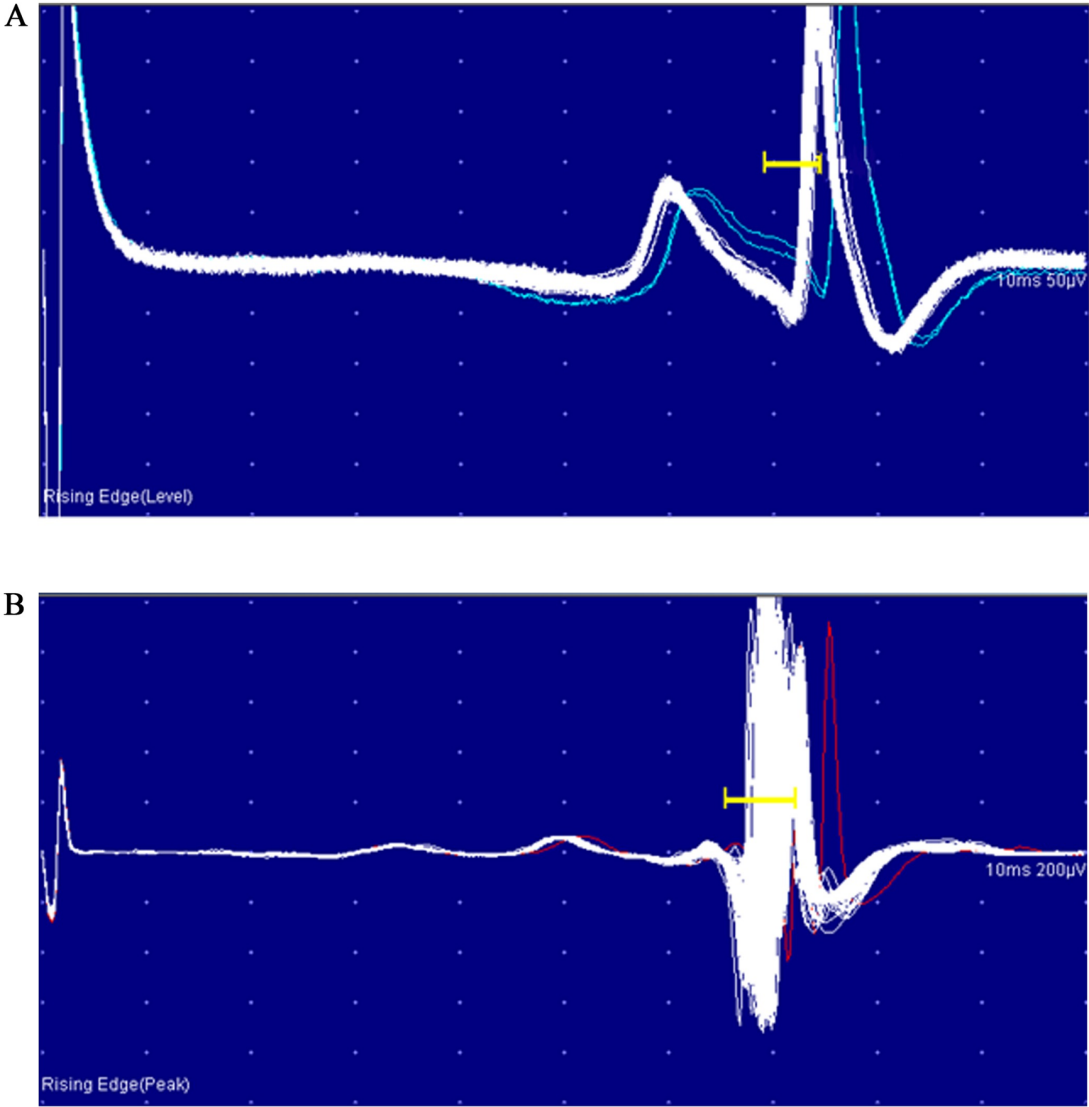
SfEMG tracings of a single fibre contraction. A. A normal SfEMG tracing of a single muscle fibre. B: Increased jitter seen in a SfEMG recording of a single muscle fibre.

**Fig 2:**
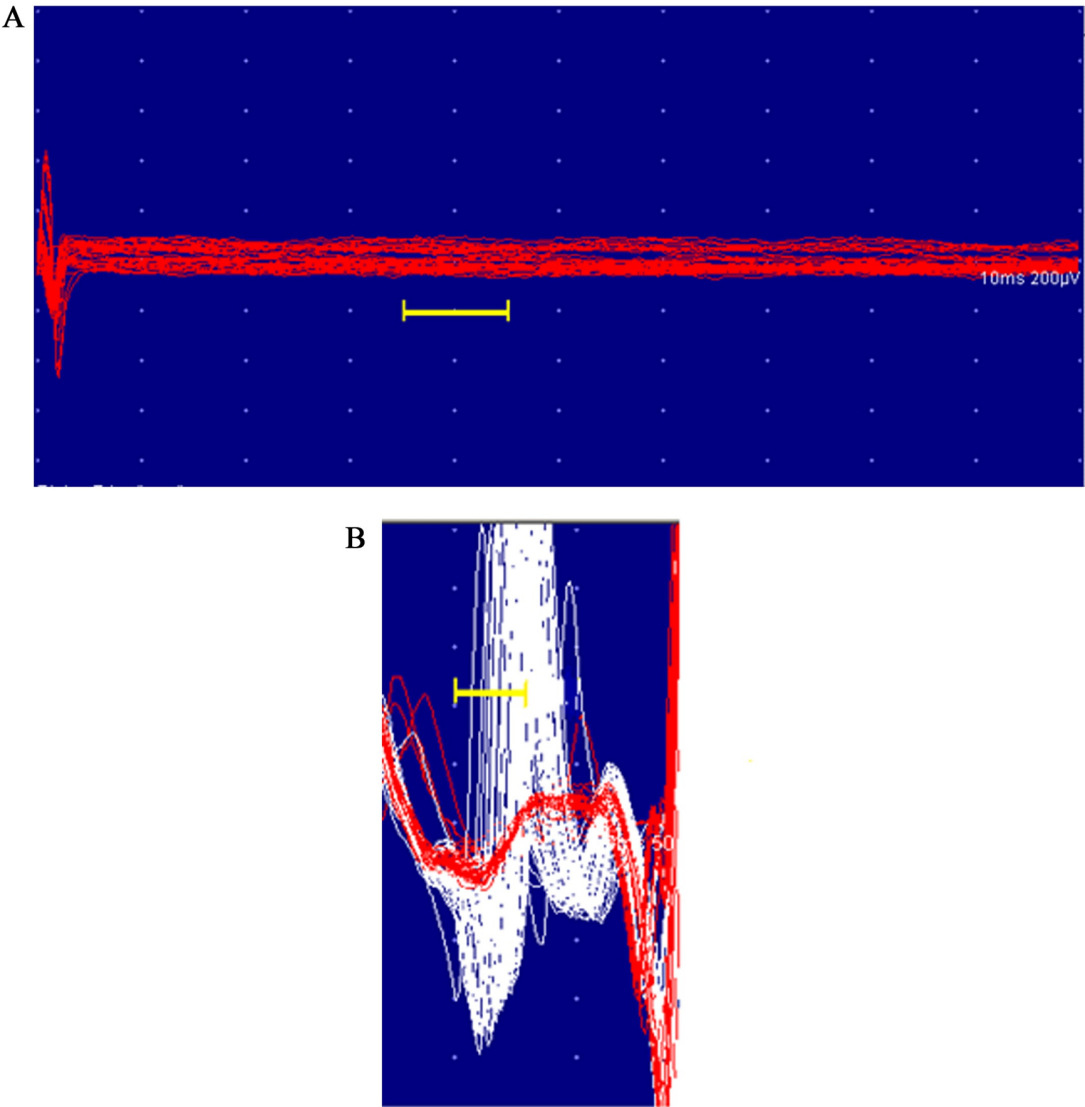
SfEMG tracings of blocking. A. Complete blocking of the neuromuscular junction seen in SfEMG; B. Incomplete blocking: white coloured SfEMG tracings represent the fibres with increased jitter and red coloured fibres present blocking.

The normal single fibre jitter recordings (Fig 1A) and the changes in the recordings with increased jitter (Fig 1B) and complete blocking (Fig 2A) and incomplete blocking (Fig 2B) are shown below.

## Data analysis

Statistical analysis of clinical and SfEMG data was done using PRISM, version 5 (GraphPad Software, Inc., La Jolla, CA). Since jitter distribution was skewed, the data are reported as medians and inter-quartile range (IQR). Where there were extremely abnormal SfEMG results with jitter above 200 μs and blocking, this was capped at 200 μs for the purpose of this analysis. The internationally accepted normal jitter value is 27–33.4 μs [23]. Therefore values over the cut off value of 33.4 μs were regarded as abnormal. Median jitter values and median blocking percentages of the IMS positive and negative groups were compared using the Mann-Whitney test. A modified Bonferroni correction procedure—the stepwise Hochberg approach—was used to test the statistical significance of pairwise comparisons of jitter values between patients who were clinically diagnosed with IMS (IMS+) and patients who did not meet the criteria of IMS (IMS-) at each day following admission [32]. This procedure ranks the p values (5 in number in this study) and tests the first (lowest p value) at 0.05/5. If it is significant, the procedure then tests the next value at 0.05/4, and if it is significant the next one at 0.05/3 and so on. Association between time for the jitter to become normalized and time for last extubation was assessed using Pearson’s correlation’s test.

## Results

**Fig 3:**
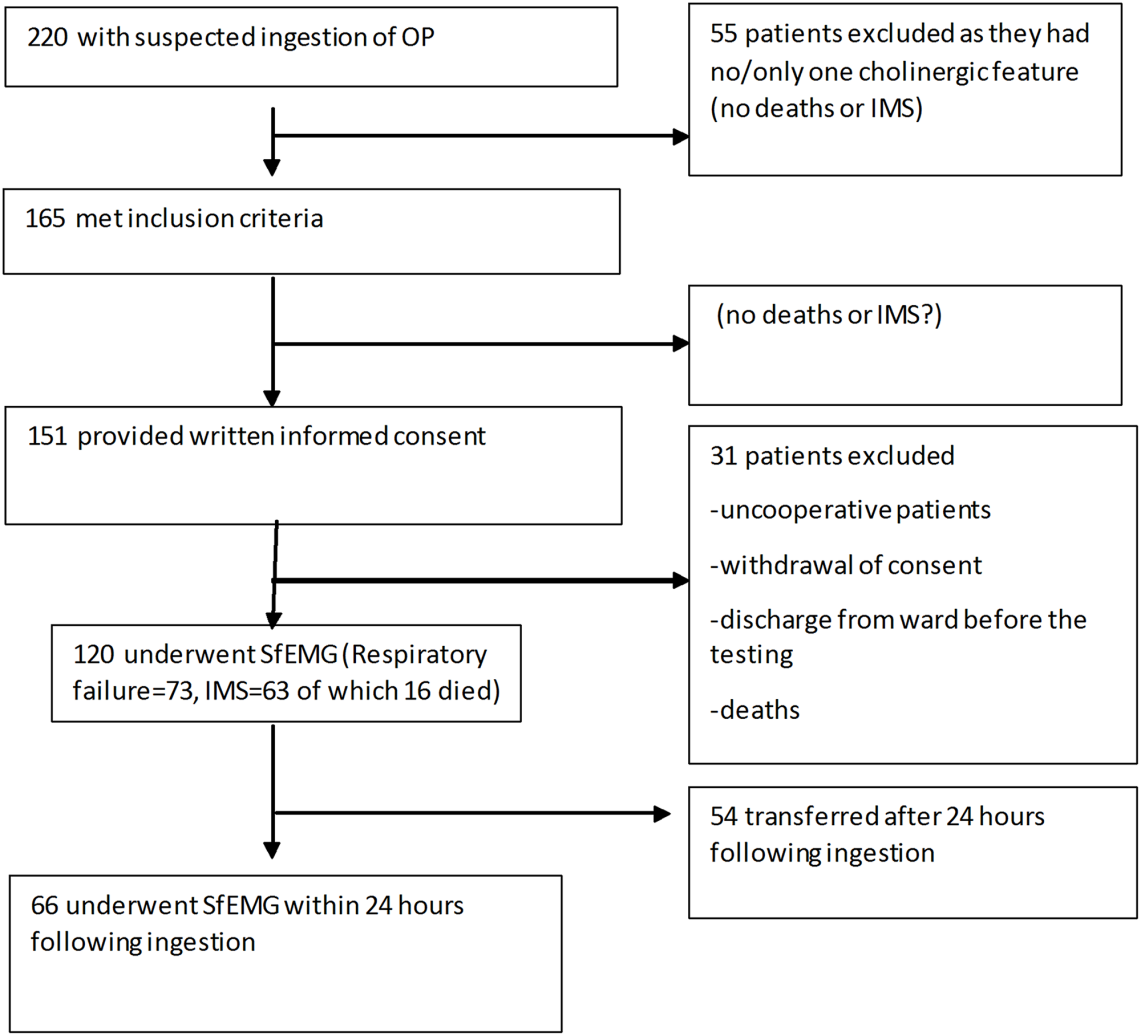
Patient recruitment flow chart.

The participant recruitment flow is shown in Fig 3. Two hundred and twenty patients attended the hospital with suspected OP ingestion. Profenofos (45%), diazinon (12%) and phenthoate (13%) were the most common agents. Of the 165 with two or more cholinergic features and reliable circumstantial evidence of OP ingestion, 151 gave informed written consent. SfEMG testing was able to be performed in 120 of these patients (Table 1); but 54 were not done within 24 hours of ingestion, mostly due to a delay in transfer to the study centre. Thus, SfEMG assessment within the first 24 hours since exposure was done in 66 patients, and these were included in the analysis for our first aim: predicting IMS using SfEMG.

**Table 1:** Actual numbers of patients who had ingested different types of organophosphates.

	IMS+	IMS- and intubated	IMS- and non-intubated.	Total evaluated
profenofos	29	24	3	53
diazinon	5	8	1	13
quinalphos	3	1	1	4
phenthoate	5	12	2	17
dimethoate	2	1	0	3
chlorpyrifos	2	3	0	5
malathion	1	0	0	1
pyrofos	0	3	0	3
fenthion	0	1	0	1
Other	-	-	-	-
unknown	16	4	3	20
**Total**	**63**	**57**	**10**	**120**

Sixty-three patients were clinically diagnosed with IMS between 24–96 hours post ingestion. Seventy three patients were intubated following respiratory failure either in the referral hospital or after admission to our hospital and this included all 63 IMS+ patients. The agents ingested by the 73 with respiratory failure, were profenofos (32), diazinon (6), quinalphos (4), phenthoate (7), dimethoate (2), chlorpyrifos (2), malathion (1) and unknown (19).

The characteristics of the patients who underwent SfMEG are shown in Table 2. The median age was 40 years (IQR = 25–52), and 98 (81%) were male. The median time from ingestion to intubation was 7.5hours (IQR = 3.5–15.4) (mean = 13.4±16.5). In the group of patients who developed IMS (IMS+), the median duration of ventilation was 161.5 hours (IQR = 78.25–259.5) while the median duration of hospital-stay was 264 hours(IQR = 192–360). These were significantly longer than the corresponding durations in the group of patients who did not develop IMS (IMS-) (p<0.05).

**Table 2:** Characteristics of the 120 patients enrolled for the SfEMG study.

	IMS+ (n = 63)	IMS- (n-57)
**Number of patients intubated**	63/63	Oct-57
**Number with SfEMG <24 hours**	28	38
**Median time from exposure to intubation, hours (IQR)**	7.5(3.5–15.4)	3.2(1.7–51.7)
**Median duration of intubation in patients, hours (IQR)**	162 (78.2–259.5)	57(41.4–135.5)
**Number of deaths (%)**	17(26.5%)	0
**Median duration of hospital-stay, hours (IQR)**	264(192–360)	72(24–108)
**Number of patients with different types of OP ingestion**		
**Profenofos**	29	24
**Phenthoate**	5	12
**Diazinon**	5	8
**Chlorpyrifos**	2	3
**Dimethoate**	2	1
**Quinalphos**	3	1
**Malathion**	1	0
**Pyrofos**	0	3
**Fenthion**	0	1
**Unknown**	16	4

Of the 120 patients who underwent SfEMG, 96 were subjected to two or more consecutive recordings. The remaining 24 patients had only a single recording as they were discharged early after recovery.

### Prediction of IMS with SfEMG jitter

Of the 66 patients who had SfEMG within 24 hours of ingestion, 28 subsequently developed IMS. Of those 28, 24 had increased jitter. Of the 38 patients who did not develop IMS 16 had increased jitter values. Prolonged jitter (>33.4μs) within the first 24 hours was associated with greatly increased risk of IMS (odds ratio = 8.9, 95% confidence intervals = 2.4–29.6, p = 0.0003; sensitivity 86%, specificity 58%).

### Changes in jitter and blocking in IMS+ and IMS- patients over time

**Fig 4:**
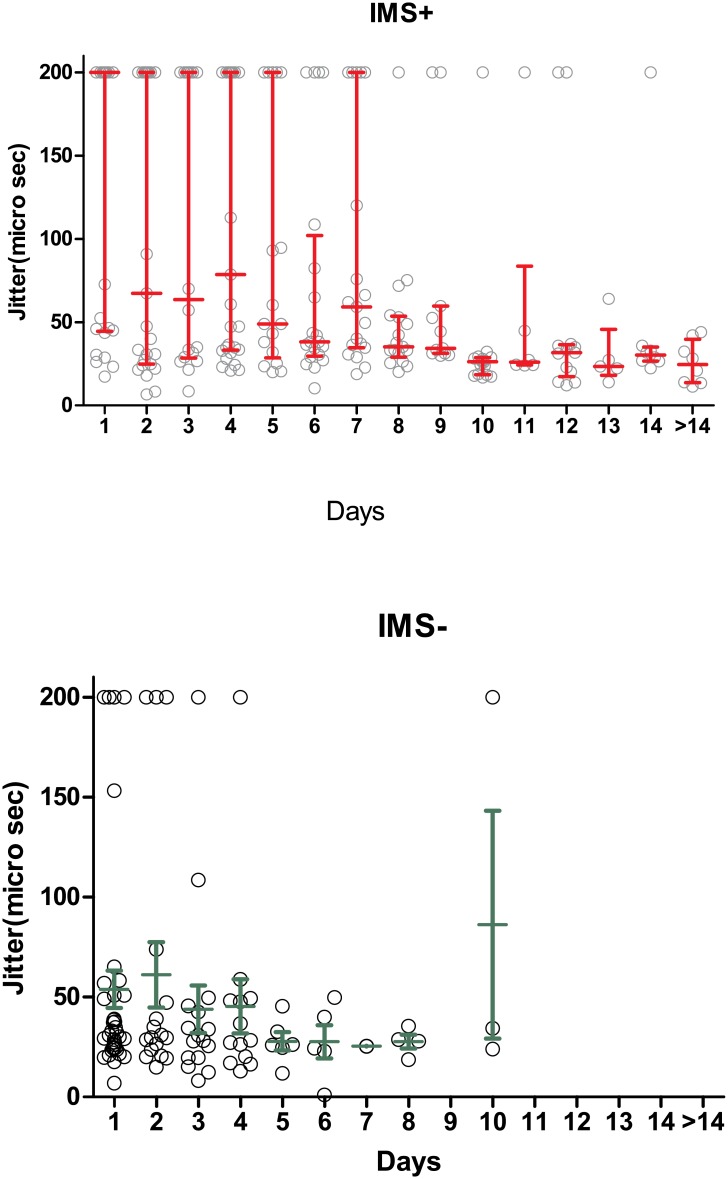
Median jitter with inter-quartile ranges in IMS+ and IMS- groups over time.

**Fig 5:**
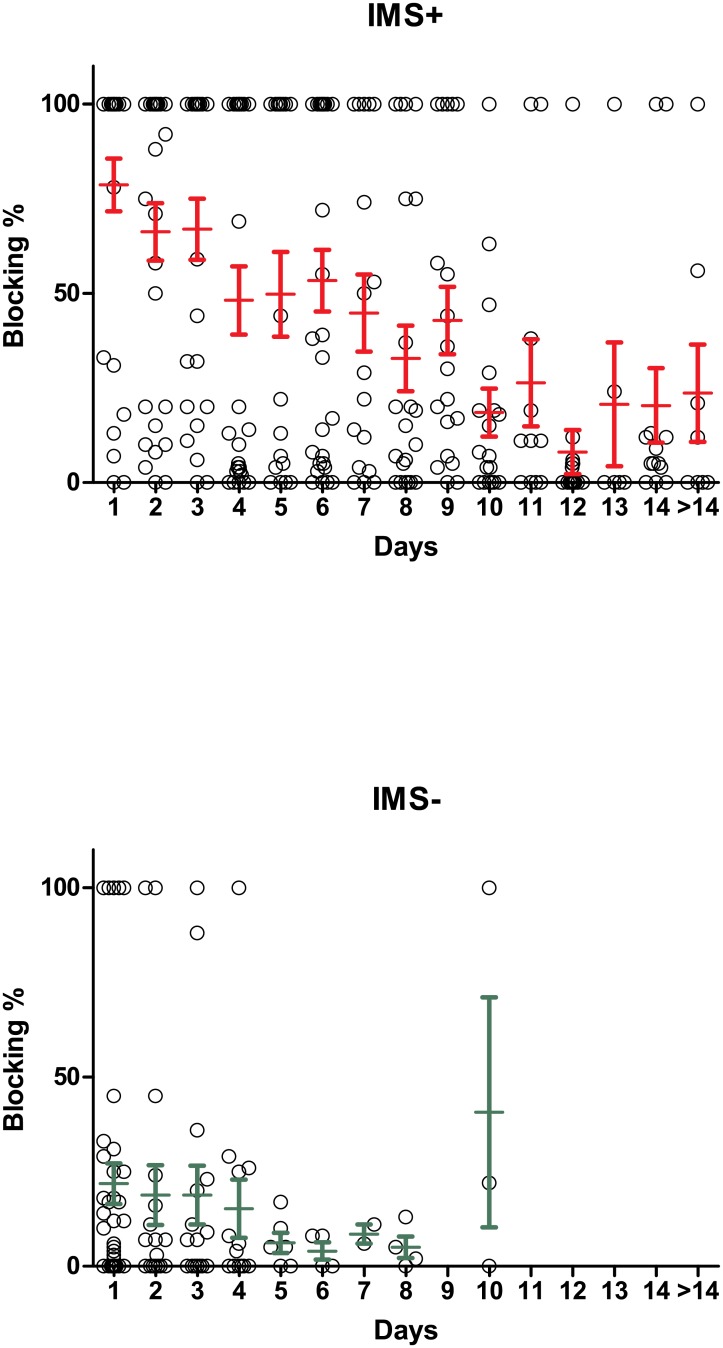
Median blocking percentages over time with inter quartile ranges in IMS+ and IMS- patients.

**Fig 6:**
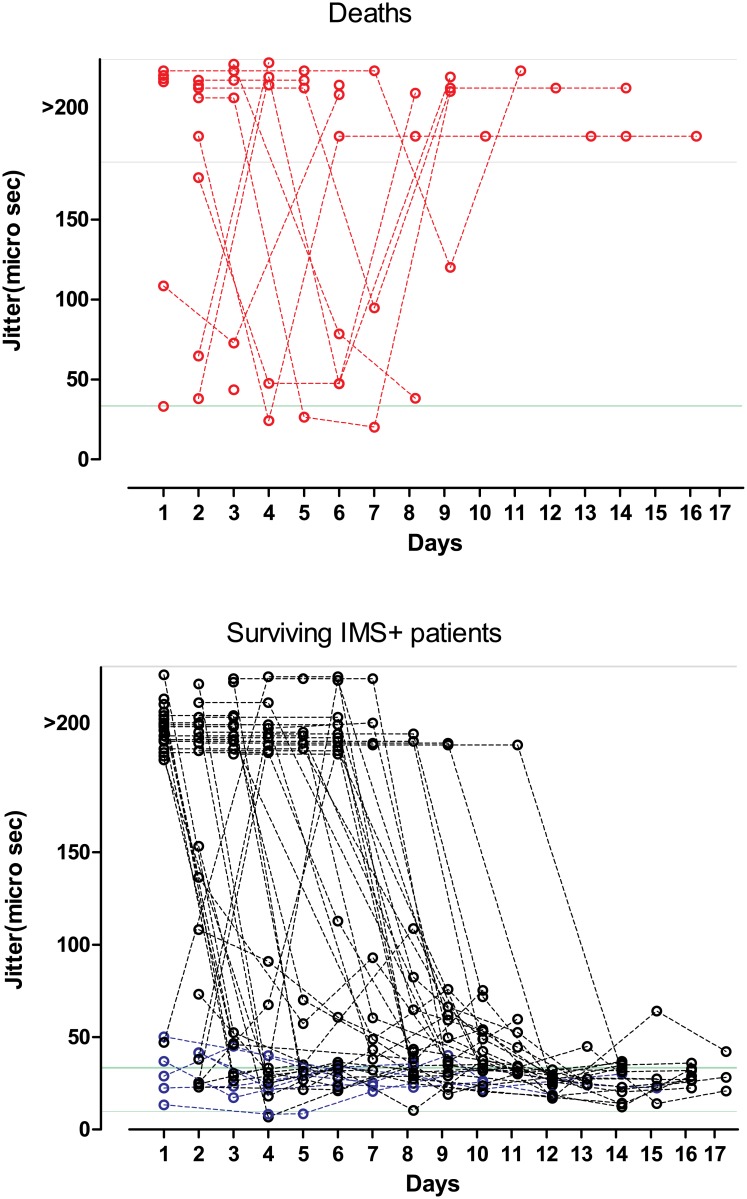
Variation of jitter over time in patients who died and those who survived with IMS over time. (Green represents the range of normal jitter and grey represents jitter >200 μs).

The changes in median jitter and median blocking percentages are shown in Figs 4, 5 and 6. In the IMS+ group the median jitter remained above >200μs initially, decline over time and became <33.4 μs on day 9. In the IMS- group, it remained more or less normal throughout the course of hospitalization (Fig 4). The IMS+ group had significantly prolonged jitter than the IMS- group at the time windows <24 hours (p< 0.0001), 24–48 hours (p = 0.006) and 48–72 hours (0.0003), but not at 72–96 hours (p = 0.10) or beyond. The lack of significant differences at later time points partly reflects the time-course of recovery of the neuromuscular junction in IMS+ patients, but also is could be a function of loss of statistical power as fewer patients (especially IMS- patients) were measured beyond this time point.

In the IMS+ group the median of fibre blocking percentage was initially high and declined over time and became closer to 5% around day 10. In the IMS- group it remained closer to normal until about day 10 (Fig 5). The IMS+ group had significantly prolonged blocking percentages than the IMS- group at the time windows <24 hours (p< 0.0001), 24–48 hours (p = 0.0004) and 48–72 hours (0.0003), and at 72–96 hours (p = 0.016), but not beyond, again partly reflecting the time course of recovery of the neuromuscular junction but also the falling numbers in the IMS- group for comparison.

There was no significant difference among the last pre-discharge median jitter values of the IMS+ group and IMS- group and healthy Sri Lankan subjects (historical unpublished data). The median jitter in the IMS+ group on discharge was 30 μs which is within the internationally accepted normal range [23]. The patients who died were excluded. However patients who self-discharged were included in the analysis.

### Red blood cell acetylcholinesterase levels (AChE)

The lower limit of normal red blood cell AChE is 25 units per gram of haemoglobin (U/gHb) [33]. On admission, all IMS+ patients had complete inhibition of red blood cell AChE (0 U/gHb) except two who had levels between 0 to 1 U/gHb. Twenty of the IMS- patients had zero levels of red blood cell AChE levels and seventeen had levels > 0 U/gHb (Median = 0 U/gHb, inter quartile range 0–2.7). The red blood cell AChE levels measured within the first 24 hours of OP ingestion were significantly lower levels in the IMS + group (Mann-Whitney U test, p = 0.001). The IMS+ patients had persistently low levels of red blood cell AChE levels even after neuromuscular junctional recovery.

### Association between time to jitter normalization and time to extubation

**Fig 7:**
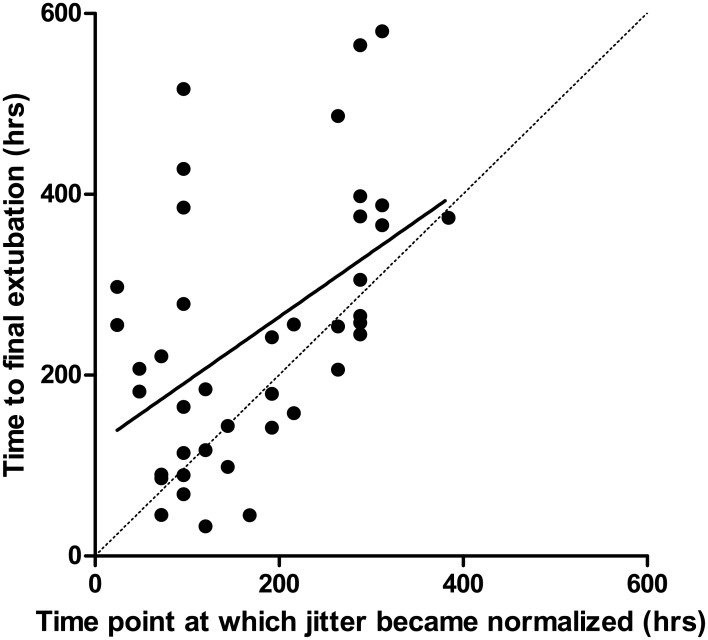
Scatter plot between the time point at which jitter became normalized and time to extubation (Pearson’s product moment correlation coefficient = 0.49, P = 0.001).

The median time for extubation from ingestion in the IMS+ group was 162 hours, which often occurred around the time that their median jitter became normal. Therefore, we plotted the earliest time point at which the jitter became normal and the time point at which the patient was extubated (Fig 7). The time for jitter to normalize had a moderate positive correlation with the time to extubate (Pearson’s r = 0.49, P = 0.001).

### Patient deaths

Sixteen patients died, all these patients were in the study and diagnosed with IMS. The compounds ingested were profenofos (11), dimethoate (2) and unknown (3). Red blood cell AChE levels were persistently depressed (<12.5 U/gHb which is 50% of lower limit of the normal range) in these patients until death. Four patients had complete blocking throughout the period. Of the remaining twelve patients, seven had persistently high jitter values throughout the course of the illness until death, four had prolonged jitter initially which dropped to a normal value mimicking apparent recovery and rising back to a complete stage of blocking before death, and the remaining one patient had normal jitter values on day 1 after admission and died on day 2 (Fig 6). All 16 patients were continuously intubated until death. The cause of death was attributed to cardio-pulmonary failure due to OP poisoning (14), pneumonia (1) and cerebro-vascular accident (1).

## Discussion

To our knowledge this is the largest clinical study on IMS to date. The key strength in our study was the ability to test 66 patients within the first 24 hours following ingestion before the onset of IMS and to use that data for predictive purposes.

Nearly one third of the patients in this cohort developed IMS. This was substantially higher than a 2008 study in Sri Lanka where 12.8% developed IMS [9]. The higher proportion that we observed is probably because the THP is a tertiary-care referral centre equipped with a specialized ICU for the management of toxicology patients, and therefore more ill patients following OP poisoning are being transferred to the unit from the periphery. The previous Sri Lankan study was conducted in a secondary care hospital. Another study from India reported that 17.6% of patients who ingested either OPs or carbamates developed IMS [14]. As carbamates are reversible inhibitors of AChE, prolonged effects such as IMS are rare [34, 35], which might have resulted in this lower incidence.

In our study we aimed to test the neuromuscular junction function in IMS [15, 27]. SfEMG characteristically shows abnormalities at the neuromuscular junction before there is apparent clinical failure of neuromuscular communication. This is seen as increased jitter or blocking in the SfEMG recording. In complete blocking (100%) there is no conduction of the impulse through the neuromuscular junction indicated by the absence of a waveform. In healthy individuals it is considered normal to have less than two fibres blocking per 20 fibres tested (<10%) [36]. In our study group we observed very high jitter values in IMS patients with blocking observed up to 100% within the first 24-hour post-exposure period before they proceeded into IMS.

We observed a median jitter of 200 μs and a median blocking percentage of 100% within the first 24 hours in the IMS+ patients. The odds of a patient with an increased jitter recorded within first 24 hours following OP ingestion developing IMS was 8.9 times that of those who did not develop IMS. The SfEMG proved to be a test with high sensitivity (86.21%), indicating the potential of the test to screen out patients who are unlikely to develop IMS, and triage other patients for close monitoring for complications such as respiratory failure and possible subsequent deaths. This could optimise the usage of the limited intensive care facilities available in countries like Sri Lanka. This could also limit unnecessary transfers of mildly poisoned OP patients to tertiary care hospitals. Overall this could improve utilisation of resources in management of OP patients. In 2009 in Sri Lanka, 70% of all poisoning management costs were utilised by patients with OP ingestion, accounting for 2.8% of the total health care budget [3]. However, the specificity of the test was poor (57.89%) making it difficult to infer a patient would develop IMS just because the SfEMG recordings are abnormal.

Recently we reviewed the utility of various clinical, biochemical and neurophysiological methods in predicting IMS [37]. A clinical study conducted in India on a mixed group of OP and carbamates reports that International Program on Chemical Safety Poison Severity Score (IPCS PSS) <2 had sensitivity of 94% and a specificity of 60% in predicting IMS [14]. In that study IMS was ascertained using more stringent criteria (i.e. presence of neck muscle weakness and proximal limb muscle weakness with or without respiratory failure) with a wider time window (i.e. between 24 hours and 7 days post ingestion). Further they administered pralidoxime to patients who presented within 24 hours with OPI ingestion. In contrast, we have used more criteria (3 out of 4) in a narrower time window to define IMS as per its original definition [9, 12]. Therefore it is difficult to directly compare the results of the two studies.

We believe that SfEMG is a more rational method to predict IMS. Prediction of IMS based on biochemical markers such as serum cholinesterase levels and red blood cell AChE levels is not useful [38–41]. In our and other studies, red blood cell AChE inhibition performed poorly; AChE levels were generally close to zero from admission onwards in all patients who had significant cholinergic features within our cohort and in most patient samples in earlier studies [37, 42]. Previous neurophysiological studies mainly focused on describing how the findings change over the course of the disease rather than prediction [9, 15–18, 20, 37].

In our study SfEMG indicated dysfunction for up to 9 to 10 days, with clear differences for IMS + patients for at least 72 hours. Thus the SfEMG is likely to be useful at any stage in triaging high-risk patients for close monitoring for IMS and potential respiratory failure.

In the IMS+ group, it took around 9 to 10 days from the time of ingestion for the SfEMG jitter to reach values of healthy population. The blocking percentage remained abnormal for up to 10 days as well. The precise pathophysiology of IMS is not known yet, but our earlier work hypothesize that in IMS acetylcholine receptors are degraded, desensitized or down-regulated by the excessive amounts of acetylcholine in the synaptic cleft of the neuromuscular junction [15]. Acetylcholine receptors of the neuromuscular junction are reproduced on average at a rate of 9% per day [43], and this rate would fit with our finding that recovery of the electrophysiology of the neuromuscular junction takes 8–9 days. Therefore our findings further supports the hypothesis that IMS is due to neuromuscular junctional block from overstimulation [15].

The median time for extubation from ingestion of the poison in the IMS+ group was 184 hours. This is close to the mean time taken for the median jitter to become normal in the same group (216 hours). Upon the above observation, we examined the correlation between the time for the jitter to become normalized and the time to final extubation, which was significant but moderate (r = 0.49). Even such moderate correlation is noteworthy given that the gross variability of the timing of our SfEMG assessments (i.e. every other day), and the non-clinical factors determining timing of extubation, for example the timing of the ICU rounds by the intensivists. These extraneous factors would be expected to reduce the strength of association between the two parameters. Therefore, the proximity of the timing of jitter normalization and that of respiratory recovery, and the correlation between the two are worthy of further exploration. We speculate such future research could explore the role of SfEMG not only in predicting onset of IMS, but also in predicting respiratory recovery thus contributing to the decision to extubate IMS patients.

Nearly eight percent of the patients in our cohort died. All these deceased had characteristic clinical features of IMS. All but one patient continued to have very high jitter values with significant blocking throughout the time they were hospitalized. Four patients were apparently recovering with jitter reducing over time and then a sudden increase in jitter was seen just before their death. Two of these four patients had ingested profenofos, which is known to cause relatively delayed effects that are consistent with the above SfEMG findings [42]. This observation suggests that it is advisable to monitor patients with profenofos poisoning for longer periods despite apparent early recovery.

In clinical settings, the neuromuscular junction is best assessed with one of two electrophysiological techniques: repetitive nerve stimulation (RNS) or SfEMG. We believe SfEMG is the better of the two methods on three accounts. First, compared to SfEMG, the abnormalities in RNS are not seen until a neuromuscular transmission is already fading. Second, RNS is usually performed on peripheral muscles in the hand which may not become weak in IMS [9, 16, 19]. Third unlike SfEMG, RNS requires patient-corporation which is only possible if the patient is able to understand the commands and voluntarily contract the tested muscles to a specific degree, meaning that a recording could not be obtained in severely affected unconscious patients. However, like RNS, SfEMG requires specialized equipment and a neurophysiologist trained in the technique. Therefore we believe that the technique would be implementable only in secondary or tertiary care hospitals in the parts of the world where OP poisoning is common.

## Supporting information

S1 File OP data for PLOS ONE. (XLSX)
